# The Influence of Mesh Density on the Results Obtained by Finite Element Analysis of Complex Bodies

**DOI:** 10.3390/ma16072555

**Published:** 2023-03-23

**Authors:** Cristian Pisarciuc, Ioan Dan, Romeo Cioară

**Affiliations:** Department of Engineering and Industrial Management, Transilvania University of Brasov, 500036 Brasov, Romania

**Keywords:** FEA simulation, mesh density, influence analysis, complex parts, elastic displacements, exact solution, numerical solution, estimation error

## Abstract

Finite element analysis of complex bodies is frequently used in design to determine the size of deformations. Successive iterations, with progressive refinement of mesh densities, are most often required to obtain a sufficiently accurate convergent numerical solution. This process is costly, time consuming, and requires superior hardware and software. The paper presents a quick and effortless way to determine a sufficiently accurate value of the numerical solution. The mentioned solution is obtained by amending the numerical solution resulting for a certain value of the mesh density of the studied body with an adequate proportionality coefficient determined following the deformation study of simple bodies differently subject to external forces. It is assumed that the elastic displacement of the various bodies has a similar evolution as the mesh density increases and that the values of the proportionality coefficients considered are approximately equal for identical mesh densities. Examples presented are related to the reference body of the mechanical press PAI 25.

## 1. Introduction

Finite element analysis (FEA) has many and various uses in engineering. For numerous applications, it is interesting to determine with sufficient precision, from the design phase, the deformation of bodies or assemblies used in various branches of engineering, mechanical engineering being one of them. In general, the finite element method is frequently used because it provides a general and systematic approach [1] regardless the area of application.

About elastic deformation of complex bodies, it is impossible to obtain the analytical solution of the exact result, but Bathe [2,3] emphasizes that this solution exists, that it is unique and, especially, that “an approximation of this exact solution can be obtained with very high accuracy using finite-element methods”. Exact analytical solutions can only be obtained when the studied bodies have quite simple geometries. For this reason, numerical methods are often used to obtain reference solutions (Hiller and Bathe) [4]. There will always be a difference between the exact solution, if it is revealed, and the approximate numerical solution.

Establishing the mesh characteristics [5] is a key step, its density determining the quality (accuracy) of the results obtained, the software and hardware requirements, the duration of the study and its cost. The reference solution must be sufficiently precise for each case. In agreement with Alwathaf [6], “the accuracy can be improved by using a finer mesh or by using refined elements”. It is known that (Liu, 2013) [7] “the accuracy of the FEA results is determined by the finite element size (mesh density)”. The use of a progressively fine-grained mesh will lead to increasingly accurate results if the finite element formulation meets the convergence criterion. It is possible to verify whether the convergence rate is uniformly optimal either by an inf-sup numerical test or by tracing the convergence to zero of the error norm (Hiller and Bathe) [4].

Liu [7] emphasizes that it is particularly important to choose the right size of elements “so that the created models will produce accurate FEA results”. This is exemplified by the study of a rectangular steel plate subject to bending. The conclusion being that, for static analysis, the finite element model ensures particularly good accuracy if the long side is discretized into 10 divisions. The approximately 1% error is determined by reference to the deformation corresponding to the finest discretization (160 divisions for each side), and not by reference to the theoretically exact deformation which, for this simple case, is easily accessible.

For a cantilever beam, Krishnamoorthi et al. [8] present yield values, using finite element analysis with different fineness of discretization, compared to the exact value determined analytically. The convergence of the numerical solution to the exact value of the analytical solution is highlighted, the difference being small when the numerical solution considers more than 1200 elements.

Hansbo and Larson [9] show, referring to plate deformation, that the type of network has a considerable influence on the convergence of the numerical solution, which it is compared with both the exact solution and the Morley approximation. Using a criss-cross mesh “the solution is robust with respect to locking”, the blocking tendency is slightly stronger in the case of an “unstructured mesh” and is evident when using directional mesh.

For the study of bodies with complex geometry, networks with particularly good resolution are used. For example, Xu Z. et al. [10] used four models with remarkably high mesh resolutions to highlight the convergence, with the number of elements being between 204,097 and 1,603,938 (the number of nodes between 80,010 and 669,044). The study showed that the difference between the maximum displacement corresponding to mesh 3 (727,474 elements and 275,399 nodes) and mesh 4 (1,603,938 elements and 669,044 nodes) is less than 5% and as a result the study did not continue for a model with even higher resolution.

Hutton [11] comparatively shows, as examples for quite simple bodies, values of the deformation obtained as a result of the exact and numerical solution (obtained using FEA) but using a network with very few elements.

For the study of strain and deformation states of a gearbox housing, also a complex body, Cojocaru et al. [12] uses networks with a high number of nodes (between 77,400 and 475,459). Maximum displacement has a variation between 0% and 2.08%, taking as a reference the result obtained for the model with the lowest resolution. The authors’ obvious conclusion is that “the variation of maximum element size does not lead to major changes in the distributions of the displacements”. Noticeably, the complexity of the studied body does not allow the determination of an exact theoretical value of the maximum deformation. It should be noted that the deviation (error) is usually determined (both for deformation and strain) based on the result obtained for the network with the best resolution, the last in the series of models studied, this being the relevant criterion for evaluating convergence.

For bodies in which one or more areas present a special interest, a much higher local density of the mesh is used, a new concept in this sense being bicameral mesh gradation (or anisotropic mesh size control) [13]. The use of anisotropic mesh, a patented method [14], is not limited to the study of solid (complex) bodies [15], but is applied in various fields, such as the study of fluid dynamics [16] (aerodynamics [17], the study of the atmosphere [18], etc.), of the viscoelasticity [19], in the determination of the natural frequencies [1], and so on.

With the increase of the computer’s performance, it is possible to use of highly efficient software packages [20], but complex, expensive, and with limited accessibility, as stated in [21], “increasing the number of nodes can improve the accuracy of the results, but at the same time, it increases the solution time and cost”. Moreover, for many complex numerical simulations, large tetrahedral anisotropic networks are used, even with billions of elements [22], which require parallel computing resources that correspond to higher costs.

## 2. Problem Description

### 2.1. Preliminary Evaluation

When designing complex parts, variously loaded, at various design stages, it is important to anticipate for areas of interest or criticality regions how they behave under a certain level of external load, for example what is their most likely elastic deformation. As an exact analytical solution is unavailable, the finite element method is used for such parts.

In the design process, the constructive solution of the part evolves, supporting various changes and additions. Implicitly, the body’s response to external load also changes. Design engineers are interested in repeatedly finding out this information, in a short time and with a high degree of confidence, with common hardware and software resources. It is preferable that the software used for FEA to be related to the software used for the design and 3D modelling of the part in question.

A professional FEA analysis conducted after the completion of the geometrical design of the complex part, to confirm that the appropriate constructive solution is chosen, is not excluded.

This article took as its starting point the design routine of the authors and does not intend to compare various FEM analyses. At the same time, the authors wanted to identify a fast method for obtaining results with practical application without changing the default FEM analysis parameters and not related to a certain software solution. The authors were interested in identifying whether and how it would be possible, using a single iteration in FEA, to obtain for a large and complex body a reliable result for elastic deformation, obtained quickly and inexpensively using common hardware and software resources.

As a preview of the final conclusions, it was found that the degree of confidence of the obtained result is dependent on the number of discretization elements and this rule also applies to geometrically simple bodies for which exact solutions are available.

If for the latter a proportionality is identified between the deformation determined analytically (the exact solution) and the one determined by FEA, then the same ratio should also exist between a presumed exact analytical solution and the one obtained by FEA for the complex geometric body, under the conditions that both the simple geometric body and the complex body share the same external load, the same volume, are made from the same material, and have the same level of discretisation. Therefore, it would be possible to obtain a realistic result of the FEA analysis for a common level of discretization by applying an appropriate proportionality coefficient determined for a simple geometric body for which an exact analytical solution exists.

### 2.2. Reference Complex Body

**Figure 1:**
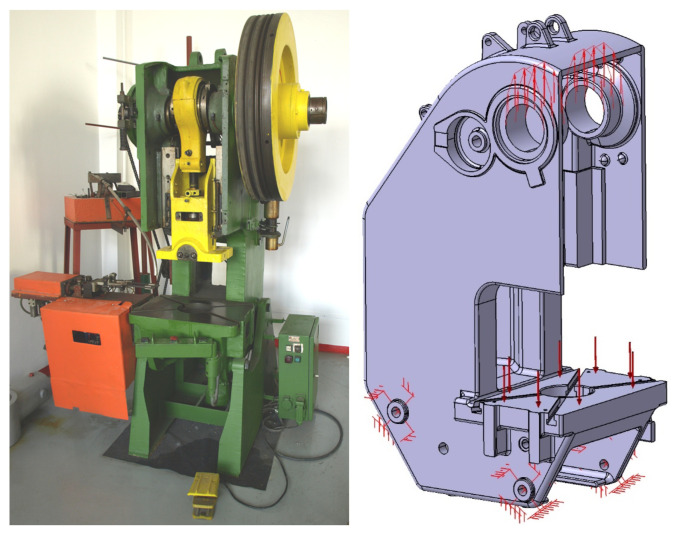
PAI 25 press and frame 3D model.

Several studies [23,24] have analysed the deformation state of a family of inclined open frames, in particular that of the single action mechanical crank press PAI 25, a cast body with complex geometry, considered here as a reference. Its virtual 3D model, shown in Figure 1, made in the ProEngineer environment, faithfully respects all the geometric characteristics of the real frame (length 580 mm, width 910 mm, height 1600 mm). The volume of embedded material is 0.144 m^3^. The frame is made of cast steel St 50-2 according to DIN, for which the following values were considered: Young’s modulus E = 210,000 N/mm^2^, shear modulus G = 80,000 N/mm^2^, and ν = 0.3 for the Poisson’s ratio.

To determine, on real conditions the deformation state, the frame was loaded with the nominal press force, *F_N_* = 250 kN, evenly distributed on the support surfaces of the main shaft bores, and on the work surface of the table, on the other hand, as shown in Figure 1. The elastic deformation was determined in the direction of the pressing axis between the bore axis of the main shaft and the plane of the workbench of the frame. For this frame, it was not possible to elaborate an analytical expression that would allow the determination of the exact solution of the elastic deformation of the frame.

**Figure 2:**
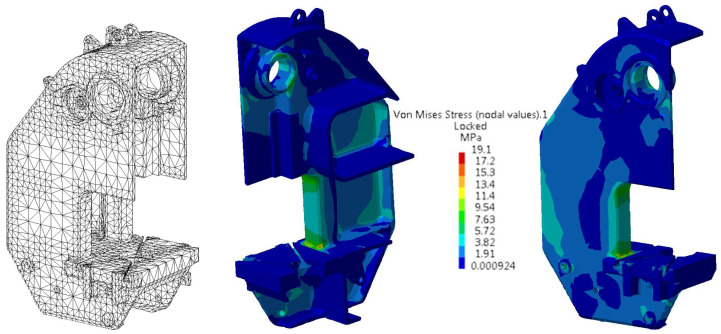
Example of 3D model discretized into tetrahedral elements and stress state for the reference body.

The finite element analysis was performed in Catia V5 R16, the software into which the 3D model made in ProEngineering was exported. The 3D model was discretized into tetrahedral elements, as shown Figure 2. The actual number of elements differs from the number of elements indicated in the program.

The study was conducted for seven levels of mesh density. The resulting values for each iteration, i.e., number of proposed and resulting elements, number of nodes, elastic deformation indicated by the program, and the deviation ζ*_i_* of deformation *y_i_* from the deviation indicated for the last iteration (*y*_7_), can be found in Table 1. In Table 2, the average size of the elements for each discretisation level are presented.

The values of the elastic deformations obtained for models with better and better resolution are ordered in a strictly ascending row. The convergence condition, stated by Bathe [2], is obviously respected, the variation of the deviation ζ*_i_* being relevant. The variation of the deformation according to the discretization levels suggests an equilateral hyperbole law.

The research conducted by the authors aimed to show that:

- a law can be established to link the deformations to the levels of the discretization;
- for various bodies, the most probable value of the elastic deformation can be determined;
- a coefficient of proportionality can be established between the theoretically exact deformation and the deformation that corresponds to a certain level of discretization, regardless of the complexity of the studied body;
- it is possible to obtain particularly good results in a short time, using common software and hardware resources, as a result of a single FEA analysis for a reasonable mesh density, related to the elastic deformation of complex geometry and large bodies.

## 3. Method and Results

### 3.1. Dependence of Deformation Values According to the Level of Discretization

As mentioned, the variation of the deformation according to the level of the discretization suggests an equilateral hyperbole law. A simple enough variation law that corresponds to the cases studied is the Equation (1)

*f*(*x*) = *a* − *b*/(*x* + *c*), (1)

where

*x*—the number of elements of the discretization network of the body subject to finite element analysis,
*y* = *f*(*x*)—the deformation of the body (under the action of the force *F*) corresponding to the discretization level to which corresponds the number *x* of elements of the network, and
$a=\underset{x\to \infty }{\lim}f\left(x\right)$ is the most probable value of the studied body deformation resulting from the action of force *F*.

To determine the values of the constants *a*, *b*, and *c*, three sets of values are required (*x*; *f*(*x*)) ≡ (*x*; *y*), in this case (*x*_1_; *y*_1_); (*x*_2_; *y*_2_), and (*x*_3_; *y*_3_).

Solving the system of Equation (2)

(2) $$\left\{\begin{array}{c} y_{1}=a-b/\left(x_{1}+c\right)\\ y_{2}=a-b/\left(x_{2}+c\right)\\ y_{3}=a-b/\left(x_{3}+c\right) \end{array}\right.$$

the expressions of the constants *a*, *b*, and *c* are obtained according to Equations (3)–(5):

(3) $$a=\frac{x_{1}y_{1}\left(y_{3}-y_{2}\right)+x_{2}y_{2}\left(y_{1}-y_{3}\right)+x_{3}y_{3}\left(y_{2}-y_{1}\right)}{y_{1}\left(x_{2}-x_{3}\right)+y_{2}\left(x_{3}-x_{1}\right)+y_{3}\left(x_{1}-x_{2}\right)},$$

(4) $$b=\frac{\left(x_{2}-x_{1}\right)\left(x_{3}-x_{1}\right)\left(x_{3}-x_{2}\right)\left(y_{2}-y_{1}\right)\left(y_{3}-y_{1}\right)\left(y_{3}-y_{2}\right)}{{\left[y_{1}\left(x_{2}-x_{3}\right)+y_{2}\left(x_{3}-x_{1}\right)+y_{3}\left(x_{1}-x_{2}\right)\right]}^{2}},$$

(5) $$c=\frac{x_{1}y_{1}\left(x_{3}-x\right)+x_{2}y_{2}\left(x_{1}-x_{3}\right)+x_{3}y_{3}\left(x_{2}-x_{1}\right)}{y_{1}\left(x_{2}-x_{3}\right)+y_{2}\left(x_{3}-x_{1}\right)+y_{3}\left(x_{1}-x_{2}\right)}.$$

Knowing the values of the constants *a*, *b*, and *c*, it is possible to determine with Equation (1) the very probable value of the deformation corresponding to any value of the number *x* of elements of the discretization network of the body undergoing finite element analysis.

When performing the study for a number *k* ≥ 3 discretization levels, $C_{k}^{3}$ combinations of value sets are available. Using Relation (3), for any set of three values (*x_i_*, *y_i_*), for the constant *a*, a value *a_r_*_-*s*-*t*_ (*r* < *s* < *t*, *r* ≥ 1, *t* ≤ *k* = *i*_max_) is determined.

For the example presented, the determination of the elastic deformation of the PAI 25 press frame in the direction of the application of technological force, performed by finite element analysis for *k* = 7 different levels of discretization, $C_{k}^{3}=C_{7}^{3}=35$ different values can be determined for the constant *a*, Table 3.

The limits *a*_min_ = 0.1824 mm and *a*_max_ = 0.23309 mm are identified, and the average value *a*_med_ = 0.19375 mm is determined. Analysing the values in Table 3 and their evolution trend, most likely *a* = *f*(*x*→∞) = 0.191 … 0.192 mm. The following values are noticeable *a*_1-2-7_ = 0.19185 mm, *a*_1-3-6_ = 0.1915 mm and *a*_1-4-6_ = 0.19183 mm, but also *a*_2-3-6_ = 0.19209 mm, *a*_2-4-6_ = 0.19206 mm, *a*_3-4-6_ = 0.19205 mm.

Obviously, none of the determined *a_r_*_-*s*-*t*_ values can be less than *y*_7_ = 0.189 mm, value of the elastic deformation indicated by the study with the greatest level of discretization. As a result, the following values should not be considered *a*_1-2-3_ = 0.18124, *a*_1-2-4_ = 0.18745, *a*_1-2-5_ = 0.18724, *a*_1-3-5_ = 0.18803, *a*_1-4-5_ = 0.18710, *a*_2-3-5_ = 0.18860, *a*_2-4-5_ = 0.18708 and *a*_3-4-5_ = 0.18666. High values that are significantly above average, such as those over 0.196 mm (namely *a*_1-5-6_ = 0.19690, *a*_2-5-6_ = 0.19802, *a*_2-5-7_ = 0.19653, *a*_3-5-6_ = 0.19987, *a*_3-5-7_ = 0.19749, *a*_3-6-7_ = 0.19537, *a*_4-5-6_ = 0.23309, *a*_4-5-7_ = 0.20598 and *a*_4-6-7_ = 0.19630), can also be excluded.

Under these conditions, the corrected average value is *a*_med-1_ = 0.192688 mm against which the new limits *a*_min-1_ = 0.19011 mm and *a*_max-1_ = 0.19588 mm deviate by −1.3379% respectively +1.6565%.

An adequate result is obtained if the results obtained for discretisation of the studied body in 5000, 18,000–20,000, and 70,000 elements is considered.

To obtain confirmation, it is necessary to perform studies on bodies with low geometric complexity for which the value of the elastic deformation can be determined analytically.

To be able to extrapolate the results, the volumes of the analysed bodies must be identical to that of the PAI 25 press frame, be made of the same material, the discretization levels must be comparable, and the external loads must be of the same value as PAI 25. In this respect, three examples are presented, chosen to differ in the nature of the general load of the bodies: compression, compression and bending only, and compression, bending, and torsion.

### 3.2. The Study of Simple Bodies in Which the Elastic Deformation Is Analytically Determinable

#### 3.2.1. Deformation Study of a Simple Pole-Type Body

**Figure 3:**
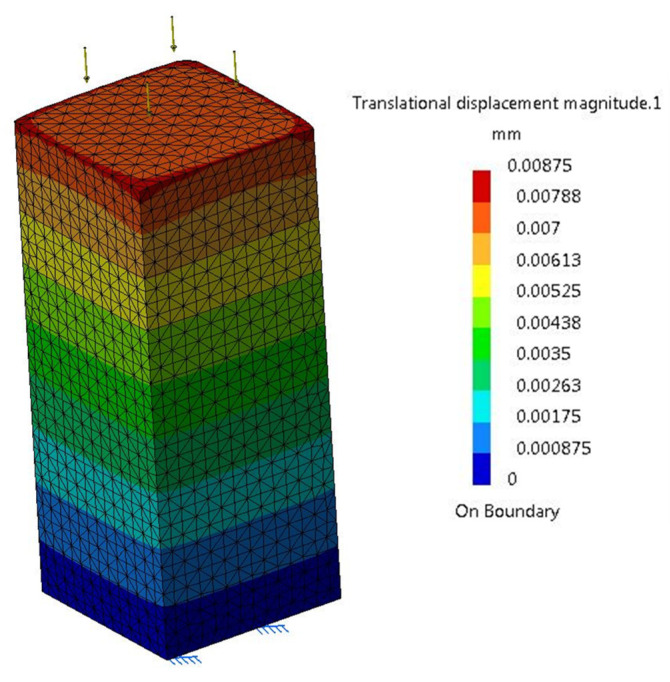
Compression pole loaded with *F* = 250 kN evenly distributed.

For a pole-type body the following are considered: length *l* = 1000 mm and section *A* = 380 × 380 = 144,400 mm^2^, and a simple compression force *F* = 250 kN evenly distributed, Figure 3.

The elastic deformation of the pole is determined analytically using the Relation (6) [25] (p. 296)

(6) $$\delta =\frac{F}{E\cdot A}\cdot l=\frac{250,000}{210,000\cdot 144,400}\cdot 1000=0.0082443\;mm.$$

Finite element analysis was performed for seven levels of levels of discretization. Similar to Table 1, the results for each study are shown in Table 4. It is noted that for all levels of discretization the elastic deformation in the direction of the resulting force is higher than the theoretically exact one, with (1.91%…7.97%).

Using Relation (3), the values for the constant *a* were calculated for all 35 combinations of three levels of discretization, Table 5. The resulting extreme values *a*_1-5-6_ = 30.211 µm and *a*_4-5-7_ = 6.9119 µm they are obviously aberrant, and value *a*_2-5-7_ = 10.003 µm is over the limit. These are not considered. The values *a*_2-5-6_ = 8.3598 µm or *a*_3-5-6_ = 8.3222 µm, both lower than any of the *y* values based on which the values of *a*_2-5-6_ are *a*_3-5-6_ were determined, cannot be accepted. The average value of the 30 acceptable values of the deformation is *a*_med_ = 8.9006 µm, and extreme values are *a*_min_ = 8.4796 µm and *a*_max_ = 9.6634 µm (which deviates from the average value by −4.73% and respectively by +8.57%). It should be noted that the average value *a*_med_ = 8.9006 µm determined this way is practically identical with the deformation *y*_7_ = 8.902 µm resulting for a discretization in 123,673 elements of the studied body (Table 4), its deviation from *y*_7_ being only +0.02%. Compared to the theoretical elastic deformation δ = 8.2443 µm, the average *a*_med_ value deviates by +7.96%.

If only reasonable values greater than the highest determined deformation are considered from Table 5, i.e., *a_r_*_-*s*-*t*_ ϵ [8.902 μm; 9.5 µm], then the corrected average value *a*_med-1_ = 9.084 µm is greater by 10.185% compared to theoretical elastic deformation δ = 8.2443 µm.

#### 3.2.2. Deformation Study of a Pole with Arm in the Console

**Figure 4:**
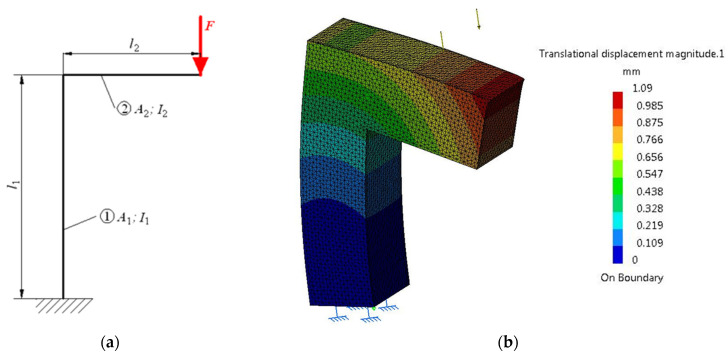
Compression and bending deformation of a pole with arm in the console. Schematic representation (**a**) and state of deformations (**b**).

A more complex load is considered in this case, compression and bending, and this is applicable to a pole with arm in the console, shown in Figure 4, loaded with a force *F* distributed linearly on the free end of the arm in the console. The pole is presented schematically through the average geometric fibre of the two elements.

To have the same volume (as the reference) of 0.144 m^3^, a square section with side *s*_2_ = 300 mm was adopted for the studied body. The relevant geometric features are lengths *l*_1_ = 950 mm and *l*_2_ = 650 mm, areas *A*_1_ = *A*_2_ = 300 × 300 = 90,000 mm^2^, with axial moments of inertia *I*_1_ = *I*_2_ = (s_2_)^4^/12 = 675 × 10^6^ mm^4^. The external force has the same value *F* = 250 kN.

The elastic deformation of the body was determined analytically using the Castigliano theorem [25] (p. 461), based on which the Relation (7) is obtained for the considered case.

(7) $$\delta =\frac{F}{E\cdot A_{1}}l_{1}+\frac{F}{E\cdot I_{1}}l_{1}\cdot l_{2}^{2}+\frac{1}{3}\cdot \frac{F}{E\cdot I_{2}}l_{2}^{3}\;=\frac{250,000\cdot 950}{210,000\cdot 90,000}+\frac{250,000\cdot 950\cdot 650^{2}}{210,000\cdot 675,000,000}+\frac{250,000\cdot 650^{3}}{3\cdot 210,000\cdot 675,000,000}\;=0.012566+0.707892+0.161449=0.881808\;\mathrm{mm}$$

The finite element analysis was performed for seven discretization levels, as in the study performed on the PAI 25 press frame. Consistent with the reasoning used in Table 1 and Table 4, the resulting values for each iteration (number of proposed and resulting elements, number of nodes, elastic deformation indicated by the program and deviation ζ*_i_* of deformation *y_i_* from the deviation indicated for the last iteration (*y*_7_)) are presented in Table 6.

Using Relation (3), the values for the constant *a* were calculated for all $C_{k}^{3}=C_{7}^{3}=35$ combinations of three levels of discretization, as shown in Table 7.

The value *a*_2-5-6_ = −0.85313 mm is aberrant, and the values *a*_3-5-6_ = 0.79783 mm and *a*_3-5-7_ = 1.17208 mm are exaggerated. These are eliminated and the average for the remaining values *a*_med_ = 0.959 mm determined. This is higher than the deformation *y*_7_ = 0.95 mm, resulting for the finest discretization of the studied body, with only +0.92%. The minimum and maximum values are *a*_min_ = a_4-5-7_ = 0.90398 mm and *a*_max_ = a_1-5-6_ = 1.05624 mm, respectively. Compared to the theoretical elastic deformation δ = 0.881808 mm ≈ 0.882 mm, the values *a*_min_, *a*_med_, and *a*_max_ deviate by +2.51%, +8.71%, and +19.78% respectively.

If only reasonable values, greater than the highest determined value, are taken from Table 7, i.e., *a_r_*_-*s*-*t*_ ϵ [0.95 mm; 1.0 mm], then the corrected average value is *a*_med-1_ = 0.9633 mm, being 9.24% higher than the theoretical elastic deformation δ = 0.882 mm.

#### 3.2.3. Deformation Study of a Pole with Double Arm in the Console

**Figure 5:**
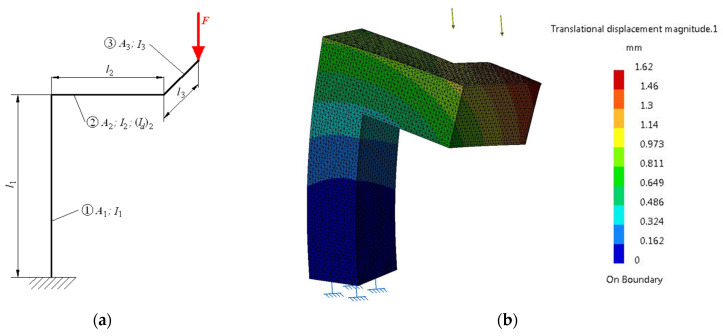
Compression, bending and torsion deformation of a pole with double arm in the console. Schematic representation (**a**) and state of deformations (**b**).

Another case of loading–compression, bending and torsion–is exemplified by a pole with double arm in the console as in Figure 5. Geometric characteristics (square section with side *s*_3_ = 280 mm, lengths *l*_1_ = 940 mm, *l*_2_ = 500 mm, and *l*_3_ = 400 mm, areas *A*_1_ = *A*_2_ = *A*_3_ = 280 × 280 = 78,400 mm^2^) ensure that the body volume is 0.144 m^3^. Axial moments of inertia are *I*_1_ = *I*_2_ = *I*_3_ = (s_3_)^4^/12 = 512.2 × 10^6^ mm^4^ and the moment of polar inertia is (*I_p_*)_2_ = (s_3_)^4^/6 = 1024.43 × 10^6^ mm^4^. The body is loaded (compression, bending and torsion) with a force *F* = 250 kN evenly distributed at the end of the arm.

The elastic deformation of the body was determined analytically using the Castigliano theorem [25] (p. 461), based on which the Relation (8) is obtained for the considered case.

(8) $$\delta =\frac{F}{E\cdot A_{1}}l_{1}+\frac{F\cdot \left(l_{2}^{2}+l_{3}^{2}\right)}{E\cdot I_{1}}l_{1}+\frac{1}{3}\cdot \frac{F}{E\cdot I_{2}}l_{2}^{3}+\frac{F\cdot l_{3}^{2}}{G\cdot {\left(I_{p}\right)}_{2}}l_{2}+\frac{1}{3}\cdot \frac{F}{E\cdot I_{3}}l_{3}^{3}\;=\frac{250,000\cdot 940}{210,000\cdot 78,400}+\frac{250,000\cdot \left(500^{2}+400^{2}\right)}{210,000\cdot 512,213,333.3}\cdot 940+\frac{250,000\cdot 500^{3}}{3\cdot 210,000\cdot 512,213,333.3}\;+\frac{250,000\cdot 400^{2}\cdot 500}{80,000\cdot 1,024,426,666.7}+\frac{250,000\cdot 400^{3}}{3\cdot 210,000\cdot 512,213,333.3}\;=0.014274+0.895739+0.096841+0.244039+0.049583=1.300476\;\mathrm{mm}$$

Once more, the finite element analysis was performed for seven discretization levels. Similar to Table 1, Table 4 and Table 6, the resulting values for each FEA analysis are presented in Table 8. It is noted that elastic deformation in the force direction determined by FEA is higher than the theoretically one, only if the level of discretization considered exceeds 63,000 elements. For the maximum level of the discretization considered, at which the number of elements is *x_i_* ≈ 100,000 elements, the value of elastic deformation indicated in the FEA analysis (*y_i_* = 1.35 mm) is 3.85% higher than the elastic deformation analytically determined using the Relation (8).

By using Relation (3), for all 35 combinations and three levels of discretisation, the values of the constant *a*, as shown in Table 9, were calculated.

Compared to the elastic deformation values obtained by FEA for the higher levels of the discretization (Table 8), the values *a*_3-5-6_ = 0.94147 mm, *a*_3-5-7_ = 0.47699 mm, *a*_4-5-6_ = 1.22024 mm, *a*_4-5-7_ = 1.18606 mm and *a*_4-6-7_ = 0.65967 mm are small, and the value of *a*_3-6-7_ = 2.40260 mm is exaggerated. By removing these values, the minimum and maximum values, *a*_min_ = *a*_3-4-5_ = 1.29288 mm, *a*_max_ = *a*_5-6-7_ = 1.57798 mm are identified, and the average value *a*_med_ = 1.37979 mm is determined. The latter is higher than the deformation *y*_7_ = 1.35 mm, resulting for the finest discretization level of the studied body, by +2.21%. The values *a*_2-5-6_ = 1.55263 mm, *a*_2-5-7_ = 1.55865 mm, *a*_2-6-7_ = 1.56498 mm, and *a*_5-6-7_ = 1.57798 mm are also unrealistically high. Excluding these values, the value of *a*_max-1_ = *a*_1-6-7_ = 1.48448 mm becomes the maximum, and the corrected average value is *a*_med-1_ = 1.35038 mm, extremely close (deviation of only +0.03%) to the deformation corresponding to the finest mesh considered in the study, *y*_7_ = 1.35 mm. Compared to the theoretical elastic deformation *δ* = 1.300476 mm, the values *a*_min_, *a*_med_, *a*_med-1_, *a*_max_, and *a*_max-1_ deviate by −0.58%, +6.10%, +3.84%, +21.34%, and +14.15%, respectively.

### 3.3. Relevant Proportionality Coefficients

Knowing the deformations *y_i_* (from Table 4, Table 6 and Table 8) obtained from the finite element analysis for each level *i* = 1 … 7 of discretization, the theoretical elastic deformation δ, the mean *a*_med_ value of the reasonable values *a_r_*_-*s*-*t*_ and an estimated value as the most probable for the deformation of the body studied under the action of the force *F*, for example *a*_med-1_ (corrected average elastic deformation), for each level *i* of discretization can be highlighted values of the proportionality coefficients (*k*_δ_)*_i_* = δ/*y_i_*, (*k*_m_)*_i_* = *a*_med_/*y_i_* and (*k_e_*)*_i_* = *a*_med-1_/*y_i_*. For the three simple cases presented, the values of the proportionality coefficients mentioned are given in Table 10, Table 11 and Table 12.

The following preliminary conclusions emerge from the analysis of the coefficients (*k*_δ_)*_i_*, (*k*_m_)*_I_*, and (*k_e_*)*_i_*. presented in Table 10, Table 11 and Table 12.

As the level of discretization increases, the values of the coefficients (*k*_δ_)*_i_* become subunit, i.e., the analytically determined deformation is smaller than the one resulting from the FEA, regardless of whether the body load is simple or more complex. For low levels of discretization, the differences between the values of the coefficients (*k*_δ_)*_i_* are relatively large regardless of whether the body is subject to simple (e.g., only compression) or more complex (e.g., compression, bending, and torsion) loads. However, for high discretization levels (10,000 elements or more), the differences between the values of the coefficients (*k*_δ_)*_i_* fade, becoming less than 4%.

Similarly, the values of the coefficients (*k*_m_)*_i_* and (*k_e_*)*_i_* decrease with the increase of the discretization level of the studied bodies, with an asymptotic variation towards 1 being evident. For low discretization levels (characterized by *x_i_* ≈ 1000 elements), the values of the coefficients (*k*_m_)*_i_* and (*k_e_*)*_i_* are significantly higher than the asymptotic limit, even by more than 40%, the magnitude of the deviation being even as the complexity of the body is rising.

Knowing values of the coefficients (*k*_δ_)*_i_*, (*k*_m_)*_I_*, and (*k_e_*)*_i_* determined according to the level of discretization of some bodies with relatively simple geometry, bodies for which it is possible to analytically determine the elastic deformation corresponding to a certain external load, it is sufficient to determine by FEA the elastic deformation for a certain discretization level to be able to estimate with sufficient precision values of interest of the respective body deformation, such as theoretical elastic deformation δ, average elastic deformation *a*_med_ or corrected average elastic deformation *a*_med-1_. They are obtained simply as a product of the value of the elastic deformation determined through FEA for the level of discretization adopted and the value of the coefficient *k*_δ_, *k*_m_, or *k_e_* corresponding to that level of discretization.

Obviously, this approach can also be applied to the PAI 25 press frame, the *y_i_* values of the elastic deformation determined using FEA for different levels of discretization being known (Table 1). The frame mentioned is subject to complex load and, as a result, the values of the coefficients (*k*_δ_)*_i_*, (*k*_m_)*_I_*, and (*k_e_*)*_i_* shown in Table 12 will be taken into account. The values thus estimated for the elastic deformation δ*_i_*, the average elastic deformation (*a*_med_)*_i_*, and the corrected average elastic deformation (*a*_med-1_)*_i_*, corresponding to each of the discretization levels are given in Table 13.

By reference to the elastic deformation δ_7_ determined for the finest discretization, to the average elastic deformation *a*_med_ and to the corrected average elastic deformation *a*_med-1_, the deviations of the values δ*_i_*, (*a*_med_)*_i_* and respectively (*a*_med-1_)*_i_*, determined using the proportionality coefficients *k*_δ_, *k*_m_, and *k_e_*, are shown in Table 14.

For discretization levels in 20,000 items or more, the deviations of all three values are becoming smaller, within a maximum range of 4%, acceptable for mainstream practical applications. For bodies with the complexity of the PAI 25 press frame, the mentioned deviations are large or much too large for discretization levels of up to 20,000 elements and as a result a study at such a level cannot be recommended. Based on the results presented in Table 14, it is recommended to determine the elastic deformation by FEA studies with mesh with 20,000-100,000 elements. Very small deviations, for all three analysed values are identified for discretization levels 5 and 6, i.e., for *x*_5_ ≈ 50,000 elements and *x*_6_ ≈ 75,000 elements respectively. These are accessible levels of discretion for common software and hardware resources and allow particularly good results in a short time in terms of the value of elastic deformation of large and complex bodies.

## 4. Conclusions

There is a great interest in determining the elastic deformation of various bodies with complex structure and varied loaded with external forces. For such bodies, most often, an exact analytical solution is not achievable, but FEA offers the possibility of obtaining a close approximate solution. However, more iterations are needed, with increasingly fine densities, which requires time, supplementary hardware, and software resources.

There are several scientific papers which, for bodies with simple geometry, show values of deformations obtained numerically compared to the exact value determined analytically. The numerical solution converges to the analytically determined value for sufficiently fine discretization levels of the studied bodies. Examples addressed in this paper only partially confirm the convergence of the numerical solution to the analytical solution. As the mesh density increases (100,000 elements or more), the numerical solution becomes larger than the analytical solution by 4–8%. Maximum differences between FEA and analytical results were obtained in the case of a simple body subjected to compression. It would have been expected that such (relatively large) differences would be identified in more complex loaded bodies, in which the analytical model took into account the geometric mean fibre and not the undeformed mean fibre.

The convergence to zero of the deviation of the numerical solution allows the highlighting of a function of variation for the values of the numerical solution depending on the level of the discretization of the investigated body. This law is one with an asymptotic increase towards the most probable value of the numerical solution of the elastic deformation related to the body under study. A value for the numerical solution is determined for each mesh density, and further analysis of such values leads to the determination of relevant values, i.e., the average value *a*_med_ of the reasonable values of the numerical solutions and the corrected average elastic deformation *a*_med-1_ (value estimated to be the most probable for deformed body studied).

For the simple geometrically studied bodies, knowing the deformations obtained from the finite element analysis for each mesh density (theoretical elastic deformation δ, mean *a*_med_ value of reasonable values and corrected average elastic deformation *a*_med-1_), the proportionality coefficients *k*_δ_, *k*_m_, and *k_e_*, were determined for each *i* level of discretization for which the study was performed. As the level of discretization increases, the values of the coefficient *k*_δ_ become subunit, i.e., the analytically determined deformation is smaller than that the one resulting from the FEA, regardless of complexity of force loading. For high mesh density (10,000 elements or more) the differences between the values of the coefficient *k*_δ_ are diminishing, becoming less than 4%. The values of coefficients *k*_m_ and *k_e_* are decreasing with the increase of the discretization level of the studied bodies, being evident an asymptotic variation towards 1.

As a future development, it will be studied whether, at least for some applications, the law of variation of the deformation’s values, according to the discretization levels proposed by Relation (1), should be replaced with a similar one in which the denominator is of higher order, e.g., the one presented in Relation (9)

*f*(*x*) = *a* − *b*/(*x*^2^ + *c*). (9)

For such a variation, the convergence towards the most probable deformation of the numerically determined values is much faster, being evident especially for discretization in a relatively small number of elements.

Knowing values of the coefficients *k*_δ_, *k*_m_, and *k_e_* determined according to the discretization level of some bodies with relatively simple geometry for which the analytical solution is easily determined, it is sufficient to determine by FEA the elastic deformation for a certain discretization level to be able to estimate with sufficient precision, by similarity, values of interest of the deformation of a complex body, such as δ, *a*_med_, or *a*_med-1_. They are obtained simply as a product of the value of the elastic deformation determined through FEA for the discretization level adopted and the value of the coefficient *k*_δ_, *k*_m_, or *k_e_* corresponding to that discretization level.

This approach was applied to the PAI 25 press frame, for which elastic deformation values were determined using FEA for different discretization levels. To determine the elastic deformation for bodies with the complexity of the PAI 25 press frame, discretization levels in 20,000 elements or more for FEA studies are recommended, levels for which the deviations of the values of the coefficients *k*_δ_, *k*_m_, and *k_e_* become small, within a maximum range of 4%, acceptable for many practical applications. Discretion levels of 20,000–100,000 elements are accessible for common software and hardware resources and allow in a reasonable time to obtain particularly reliable results for the elastic deformation of large and geometrically complex bodies.

## Figures and Tables

**Table 1:** Values obtained at the study by finite element analysis of the PAI 25 press frame.

No. (*i*)	No. of Proposed Items (Required)	No. of Nodes	No. of Resulted Elements, (*x_i_*)	Indicated Elastic Deformation, (*y_i_*) [mm]	Deviation, ζ*_i_* = [(*y*_7_ − *y_i_*)/*y*_7_] × 100, [%]
1	1000	851	2309	0.0937	50.504
2	5000	1731	5007	0.14	25.926
3	10,000	3258	9810	0.16	15.344
4	20,000	6744	22,182	0.176	6.878
5	50,000	13,925	48,728	0.182	3.370
6	75,000	20,957	76,129	0.187	1.058
7	100,000	27,721	103,410	0.189	0

**Table 2:** Average size of the elements.

No. of Proposed Elements	1000	5000	10,000	20,000	50,000	75,000	100,000
Average size of the elements, [mm^3^]	144	28.8	14.4	7.2	2.88	1.92	1.44

**Table 3:** The set of *a_r_*_-*s*-*t*_ values of the probable theoretical elastic deformation of the PAI 25 press frame, determined based on *k* = 7 FEA analyses with different discretization levels (in mm).

*a*_1-2-3_ = 0.18124	*a*_1-3-4_ = 0.19011	*a*_1-4-6_ = 0.19183	*a*_2-3-4_ = 0.19222	*a*_2-4-6_ = 0.19206	*a*_3-4-5_ = 0.18666	*a*_3-6-7_ = 0.19537
*a*_1-2-4_ = 0.18745	*a*_1-3-5_ = 0.18803	*a*_1-4-7_ = 0.19283	*a*_2-3-5_ = 0.18860	*a*_2-4-7_ = 0.19305	*a*_3-4-6_ = 0.19205	*a*_4-5-6_ = 0.23309
*a*_1-2-5_ = 0.18724	*a*_1-3-6_ = 0.19150	*a*_1-5-6_ = 0.19690	*a*_2-3-6_ = 0.19209	*a*_2-5-6_ = 0.19802	*a*_3-4-7_ = 0.19310	*a*_4-5-7_ = 0.20598
*a*_1-2-6_ = 0.19074	*a*_1-3-7_ = 0.19246	*a*_1-5-7_ = 0.19588	*a*_2-3-7_ = 0.19294	*a*_2-5-7_ = 0.19653	*a*_3-5-6_ = 0.19987	*a*_4-6-7_ = 0.19630
*a*_1-2-7_ = 0.19185	*a*_1-4-5_ = 0.18710	*a*_1-6-7_ = 0.19487	*a*_2-4-5_ = 0.18708	*a*_2-6-7_ = 0.19511	*a*_3-5-7_ = 0.19749	*a*_5-6-7_ = 0.19370

**Table 4:** Values obtained at FEA study at compression of simple parallelepiped pole body.

No. (*i*)	No. of Proposed Items (Required)	No. of Nodes	No. of Resulted Elements, (*x_i_*)	Indicated Elastic Deformation, (*y_i_*) [µm]	Deviation, ζ*_i_* = [(*y*_7_ − *y_i_*)/*y*_7_] × 100, [%]
1	1000	296	1014	8.402	5.617
2	5000	791	3153	8.588	3.527
3	10,000	2243	8921	8.592	3.482
4	20,000	4855	20,400	8.672	2.584
5	50,000	12,746	55,517	8.744	1.775
6	75,000	16,724	69,660	8.831	0.798
7	100,000	26,878	123,673	8.902	0

**Table 5:** The set of *a_r_*_-*s*-*t*_ values of the probable theoretical elastic deformation at the compression of a loaded pole, determined based on *k* = 7 FEA analyses with different discretization levels (in µm).

*a*_1-2-3_ = 8.5935	*a*_1-3-4_ = 8.7823	*a*_1-4-6_ = 8.9604	*a*_2-3-4_ = 8.5787	*a*_2-4-6_ = 9.3085	*a*_3-4-5_ = 8.8074	*a*_3-6-7_ = 9.0575
*a*_1-2-4_ = 8.6880	*a*_1-3-5_ = 8.7977	*a*_1-4-7_ = 8.9972	*a*_2-3-5_ = 8.5459	*a*_2-4-7_ = 9.1666	*a*_3-4-6_ = 9.0372	*a*_4-5-6_ = 8.5925
*a*_1-2-5_ = 8.7561	*a*_1-3-6_ = 8.9150	*a*_1-5-6_ = 30.211	*a*_2-3-6_ = 8.5360	*a*_2-5-6_ = 8.3598	*a*_3-4-7_ = 9.0476	*a*_4-5-7_ = 6.9119
*a*_1-2-6_ = 8.8498	*a*_1-3-7_ = 8.9653	*a*_1-5-7_ = 9.1949	*a*_2-3-7_ = 8.4796	*a*_2-5-7_ = 10.003	*a*_3-5-6_ = 8.3222	*a*_4-6-7_ = 9.0600
*a*_1-2-7_ = 8.9174	*a*_1-4-5_ = 8.8030	*a*_1-6-7_ = 9.0352	*a*_2-4-5_ = 8.8574	*a*_2-6-7_ = 9.0784	*a*_3-5-7_ = 9.6634	*a*_5-6-7_ = 8.9449

**Table 6:** Deformation values obtained at compression and bending study of a pole with arm in the console by using finite element analysis.

No. (*i*)	No. of Proposed Items (Required)	No. of Nodes	No. of Resulted Elements, (*x_i_*)	Indicated Elastic Deformation, (*y_i_*) [mm]	Deviation, ζ*_i_* = [(*y*_7_ − *y_i_*)/*y*_7_] × 100, [%]
1	1000	338	1099	0.74	22.105
2	5000	1307	4922	0.867	8.737
3	10,000	2696	10,804	0.891	6.211
4	20,000	5129	21,575	0.919	3.263
5	50,000	13,289	58,786	0.926	2.526
6	75,000	17,143	74,515	0.944	0.632
7	100,000	22,519	100,429	0.95	0

**Table 7:** The set of *a_r_*_-*s*-*t*_ values of the probable theoretical elastic deformation at the compression and torsion loaded of a pole with arm in the console, determined based on k = 7 FEA analyses with different discretization levels (in mm).

*a*_1-2-3_ = 0.9121	*a*_1-3-4_ = 0.9549	*a*_1-4-6_ = 0.9557	*a*_2-3-4_ = 1.0103	*a*_2-4-6_ = 0.9577	*a*_3-4-5_ = 0.9287	*a*_3-6-7_ = 0.9728
*a*_1-2-4_ = 0.9378	*a*_1-3-5_ = 0.9352	*a*_1-4-7_ = 0.9599	*a*_2-3-5_ = 0.9388	*a*_2-4-7_ = 0.9619	*a*_3-4-6_ = 0.9558	*a*_4-5-6_ = 0.9141
*a*_1-2-5_ = 0.9323	*a*_1-3-6_ = 0.9555	*a*_1-5-6_ = 1.0562	*a*_2-3-6_ = 0.9637	*a*_2-5-6_ = −0.853	*a*_3-4-7_ = 0.9605	*a*_4-5-7_ = 0.904
*a*_1-2-6_ = 0.951	*a*_1-3-7_ = 0.9593	*a*_1-5-7_ = 0.9957	*a*_2-3-7_ = 0.966	*a*_2-5-7_ = 1.0422	*a*_3-5-6_ = 0.7978	*a*_4-6-7_ = 0.9798
*a*_1-2-7_ = 0.9556	*a*_1-4-5_ = 0.9301	*a*_1-6-7_ = 0.9691	*a*_2-4-5_ = 0.9298	*a*_2-6-7_ = 0.972	*a*_3-5-7_ = 1.1721	*a*_5-6-7_ = 0.9561

**Table 8:** Values obtained at FEA study at compression, bending and torsion of a pole with double arm in the console.

No. (*i*)	No. of Proposed Items (Required)	No. of Nodes	No. of Resulted Elements, (*x_i_*)	Indicated Elastic Deformation, (*y_i_*) [mm]	Deviation, ζ*_i_* = [(*y*_7_ − *y_i_*)/*y*_7_] × 100, [%]
1	1000	324	1027	0.953	29.407
2	5000	1285	4857	1.15	14.815
3	10,000	2730	10,673	1.23	8.889
4	20,000	4937	20,097	1.26	6.667
5	50,000	12,271	50,772	1.28	5.185
6	75,000	17,364	75,224	1.32	2.222
7	100,000	22,653	99,194	1.35	0

**Table 9:** The set of *a_r_*_-*s*-*t*_ values of the probable theoretical elastic deformation at the compression, bending and torsion of a pole with double arm in the console determined based on *k* = 7 FEA analyses with different discretization levels (in mm).

*a*_1-2-3_ = 1.3311	*a*_1-3-4_ = 1.2983	*a*_1-4-6_ = 1.3466	*a*_2-3-4_ = 1.2931	*a*_2-4-6_ = 1.3502	*a*_3-4-5_ = 1.2929	*a*_3-6-7_ = 2.4026
*a*_1-2-4_ = 1.3101	*a*_1-3-5_ = 1.2948	*a*_1-4-7_ = 1.3802	*a*_2-3-5_ = 1.2936	*a*_2-4-7_ = 1.3874	*a*_3-4-6_ = 1.3668	*a*_4-5-6_ = 1.2202
*a*_1-2-5_ = 1.2991	*a*_1-3-6_ = 1.3387	*a*_1-5-6_ = 1.4416	*a*_2-3-6_ = 1.3392	*a*_2-5-6_ = 1.5526	*a*_3-4-7_ = 1.4168	*a*_4-5-7_ = 1.1861
*a*_1-2-6_ = 1.3381	*a*_1-3-7_ = 1.3697	*a*_1-5-7_ = 1.4619	*a*_2-3-7_ = 1.3719	*a*_2-5-7_ = 1.5587	*a*_3-5-6_ = 0.9415	*a*_4-6-7_ = 0.6597
*a*_1-2-7_ = 1.3671	*a*_1-4-5_ = 1.2938	*a*_1-6-7_ = 1.4845	*a*_2-4-5_ = 1.2929	*a*_2-6-7_ = 1.565	*a*_3-5-7_ = 0.477	*a*_5-6-7_ = 1.578

**Table 10:** Values of the proportionality coefficients (*k*_δ_)*_i_*, (*k*_m_)*_i_* and (*k_e_*)*_i_* for a column (simple geometric body) subject to compression (δ = 8.2443 µm; *a*_med_ = 8.9006 µm; *a*_med-1_ = 9.084 µm).

*i*	1	2	3	4	5	6	7
*y*_*i*_ (in µm)	8.402	8.588	8.592	8.672	8.744	8.831	8.902
(*k*_δ_)_*i*_ = δ/*y*_*i*_	0.9812	0.9600	0.9595	0.9507	0.9429	0.9336	0.9261
(*k*_m_)_*i*_ = *a*_med_/*y*_*i*_	1.0593	1.0364	1.0359	1.0264	1.0179	1.0079	0.9998
(*k*_*e*_)_*i*_ = *a*_med-1_/*y*_*i*_	1.0812	1.0578	1.0573	1.0475	1.0389	1.0286	1.0204

**Table 11:** Values of the proportionality coefficients (*k*_δ_)*_i_*, (*k*_m_)*_i_* and (*k_e_*)*_i_* for a pole with arm in the console, subject to compression and torsion (δ = 8.2443 µm; *a*_med_ = 8.9006 µm; *a*_med-1_ = 9.084 µm).

*i*	1	2	3	4	5	6	7
*y*_*i*_ (in mm)	0.74	0.867	0.891	0.919	0.926	0.944	0.95
(*k*_δ_)_*i*_ = δ/*y*_*i*_	1.1916	1.0171	0.9897	0.9595	0.9523	0.9341	0.9282
(*k*_m_)_*i*_ = *a*_med_/*y*_*i*_	1.2959	1.1061	1.0763	1.0435	1.0356	1.0159	1.0095
(*k*_*e*_)_*i*_ = *a*_med-1_/*y*_*i*_	1.3018	1.1111	1.0811	1.0482	1.0403	1.0204	1.0140

**Table 12:** Values of the proportionality coefficients (*k*_δ_)*_i_*, (*k*_m_)*_i_* and (*k_e_*)*_i_* for a pole with double arm in the console, subject to compression, bending and torsion (δ = 8.2443 µm; *a*_med_ = 8.9006 µm; *a*_med-1_ = 9.084 µm).

*i*	1	2	3	4	5	6	7
*y*_*i*_ (in mm)	0.953	1.15	1.23	1.26	1.28	1.32	1.35
(*k*_δ_)_*i*_ = δ/*y*_*i*_	1.36461	1.13085	1.05730	1.03212	1.01600	0.98521	0.96332
(*k*_m_)_*i*_ = *a*_med_/*y*_*i*_	1.44784	1.19982	1.12178	1.09507	1.07796	1.04530	1.02207
(*k*_*e*_)_*i*_ = *a*_med-1_/*y*_*i*_	1.41698	1.17424	1.09787	1.07173	1.05498	1.02302	1.00028

**Table 13:** Estimated values δ*_i_*, (*a*_med_)*_i_* and (*a*_med-1_)*_i_*, for PAI 25 press frame determined through amendment of elastic deformation *y_i_* with the values of proportional coefficients (*k*_δ_)*_i_*, (*k*_m_)*_i_* and (*k_e_*)*_i_*.

*i*	*y*_*i*_	(*k*_δ_)_*i*_	(*k*_m_)_*i*_	(*k*_e_)_*i*_	δ_*i*_ = *y*_*i*_·(*k*_δ_)_*i*_	(*a*_med_)_*i*_ = y_*i*_·(k_m_)_*i*_	(*a*_med-1_)_*i*_ = *y*_*i*_·(*k*_e_)_*i*_
	[mm]	-	-	-	[mm]	[mm]	[mm]
1	0.0937	1.36461	1.44784	1.41698	0.127864	0.135663	0.132771
2	0.14	1.13085	1.19982	1.17424	0.158319	0.167975	0.164394
3	0.16	1.05730	1.12178	1.09787	0.169168	0.179485	0.175659
4	0.176	1.03212	1.09507	1.07173	0.181653	0.192732	0.188624
5	0.182	1.01600	1.07796	1.05498	0.184912	0.196189	0.192006
6	0.187	0.98521	1.04530	1.02303	0.184234	0.195471	0.191305
7	0.189	0.96332	1.02207	1.00028	0.182067	0.193171	0.189053

**Table 14:** Deviations of estimated values δ*_i_*, (*a*_med_)*_i_* and (*a*_med-1_)*_i_* from values δ_7_, *a*_med_ and *a*_med-1_ for PAI 25 press frame.

*i*	Δδ_*i*_ = (*y*_7_ − δ_*i*_)/*y*_7_·100%	Δ(*a*_med_)_*i*_ = ((*a*_med_ − (*a*_med_)_*i*_)/*a*_med_·100%	Δ(*a*_med-1_)_*i*_ = ((*a*_med-1_ − (*a*_med-1_)_*i*_)/*a*_med-1_·100%
1	32.347	29.981	31.095
2	16.233	13.303	14.684
3	10.493	7.363	8.837
4	3.887	0.525	2.109
5	2.163	−1.259	0.354
6	2.521	−0.888	0.718
7	3.668	0.299	1.887

## Data Availability

The dataset of the current study is available only for the review of the present article.

## Abbreviations

*x*_*i*_	effective number of discretisation items, for discretization level *i*
*y*_*i*_	the indicated elastic deformation (numerical solution), for level *i* of discretization
*i*_max_	*i* level for maximum discretisation
*a*_*r*-*s*-*t*_ (*r* < *s* < *t*, *r* ≥ 1, t ≤ k = i_max_)	any value of the probable theoretical elastic deformation determined based on a presumed law of variation
*a*_min_, *a*_max_, *a*_med_	the minimum, maximum and average values for ^a^_^r^-^s^-^t^_ set of values
*a*_min-1_, *a*_max-1_, *a*_med-1_	minimum, maximum, and corrected average value (estimated value as the most probable) resulting from disregarding values *a*_*r*-*s*-*t*_ that are aberrant and unrealistic
δ	analytically determined theoretical deformation
(*k*_δ_)_*i*_, (*k*_m_)_*i*_, (*k*_e_)_*i*_	proportionality coefficient for the theoretical elastic deformation δ determined analytically, for the mean *a*_med_ value of the numerical solutions *a*_*r*-*s*-*t*_, and respectively for the mean *a*_med-1_ value of the numerical solutions *a*_*r*-*s*-*t*_, for discretization level *i*
ζ_*i*_	deviation for discretization level *i*
*k*	maximum level of discretization
